# Ptomaines and Other Animal Alkaloids

**Published:** 1893-03

**Authors:** 


*Ptomaines and other Animal Alkaloids.* By A. C. FARQUHARSON, M.D. Pp. xi., 152. Bristol: JOHN WRIGHT & CO. 1892.

Within the compass of 138 pages the author has endeavoured to give a history of animal alkaloids, with an account of their chief chemical properties, their mode of formation, their action on the human body, and the methods by which they may be detected and prepared. He explains in the preface that the book "lays no claims to originality. An attempt is made to bring together in an accurate and concise *résumé* the main facts pertaining to a subject which does not receive the attention in this country that it deserves." The subject is such a complex and difficult one that a great deal of elucidation is required by the ordinary reader, and this is not sufficiently given; the book is, in fact, *too* concise. It cannot even be considered a complete *résumé*, for within such narrow limits this is impossible.

The methods of extraction are well summarised, and the clinical features of acute and chronic ptomaine poisoning are clearly told. On the supposition, however, that "a pathological state is one of exaggerated function," some "explanations" are given which will hardly be accepted by many. Some interesting cases are recorded, and the effects of these alkaloids on the nervous system are discussed at some length.

To the physiological chemist the book will appear a somewhat imperfect summary; to the majority of readers, who start with no clear knowledge on the subject, the want of further explanation will be a drawback; but great care has evidently been bestowed on the work, and we can hardly expect to have the vague and loose knowledge which has been obtained on these bodies made clear and definite in so small a volume. There is a copious index, and the type is good.

